# Osteoporosis management in Australian aged care facilities: a mixed method study

**DOI:** 10.1007/s11657-024-01401-7

**Published:** 2024-05-14

**Authors:** Catherine Laird, Kylie A. Williams, Helen Benson

**Affiliations:** Graduate School of Health, University of Technology Sydney, PO Box 123, Sydney, NSW 2007 Australia

**Keywords:** Osteoporosis, Aged care, Medication review, Pharmacists

## Abstract

**Summary:**

Osteoporosis is a common but sub-optimally managed disease amongst aged care residents. Pharmacists undertaking comprehensive medication reviews is one strategy to improve osteoporosis management. Analysis of pharmacist medication review recommendations has identified common clinical practice issues that can be addressed to optimise osteoporosis management for aged care residents.

**Purpose:**

This study investigates the prevalence of osteoporosis medicine use amongst Australian aged care residents and explores drug-related problems (DRPs) identified during medication reviews and pharmacist recommendations to resolve them.

**Methods:**

Resident demographics, medications, diagnoses, osteoporosis related DRPs, and recommendations to resolve them were extracted from medication review reports. A mixed methods approach was taken to analysis, involving descriptive statistical analysis and content analysis.

**Results:**

Medication review reports relating to 980 residents were collected. Antiresorptive therapies were used by 21.7% of residents, of which 87.2% were prescribed denosumab. Osteoporosis related DRPs represented 14.0% of all DRPs identified by pharmacists. Vitamin D was involved in 55.4% of these DRPs, the remainder concerned antiresorptive therapies (23.4%), medications contributing to osteoporosis (16.3%), and calcium (4.9%). Frequent deviations in practice from aged care clinical guidelines and consensus recommendations concerning vitamin D and calcium were found. DRPs and accompanying recommendations relating to denosumab revealed inadequate monitoring and inadvertent therapy disruptions.

**Conclusion:**

Pharmacist identified DRPs and recommendations revealed common aspects of clinical practice that can be addressed to improve osteoporosis management for aged care residents. A need to raise awareness of aged care-specific consensus recommendations concerning vitamin D and calcium is evident. Facility protocols and procedures must be developed and implemented to ensure safe and effective use of denosumab.

## Introduction

Osteoporosis is the most prevalent metabolic disease affecting the elderly [1]. It is estimated that over 80% of aged care residents live with osteoporosis and that they sustain osteoporotic hip fractures at a rate of up to four times that of community-dwelling individuals [2, 3]. Fractures resulting from osteoporosis reduce the quality and length of life for aged care residents and place increased pressure on care providers [2–4]. A wide body of evidence supports the use of vitamin D, calcium, and antiresorptive therapies, with safety and effectiveness demonstrated for aged care residents [4]. Despite this, osteoporosis management is sub-optimal amongst the aged care population [5–9].

Several reasons for the sub-optimal management of osteoporosis in aged care have been proposed [4, 5, 10, 11]. These include a change of healthcare providers on admission to the aged care facility, a strong emphasis on deprescribing rather than the commencement of therapy, and difficulties in diagnostic testing in this population [4, 5, 10, 11].

Residents commonly experience a change of healthcare providers upon entry to residential aged care. An Australian study reported 72.2% of residents had a change of general practitioner upon admission, whilst a Canadian study similarly reported that only 12.1% of residents retained their family physician [12, 13]. Receipt of incomplete medical histories by the new care team can lead to disruptions in osteoporosis management [10, 14].

The multiple comorbidities and frailty of aged care residents make them highly susceptible to adverse drug reactions, leading to a reported emphasis on deprescribing opportunities [15]. This focus on deprescribing, rather than prescribing, is linked to a hesitance to investigate and commence therapy for chronic conditions such as osteoporosis [10, 11]. Treatment commencement is further impeded by the logistical challenges associated with undertaking bone mineral density (BMD) testing using dual-energy X-ray absorptiometry (DEXA) for aged care residents, resulting in underdiagnosis [10, 11, 16]. This gives rise to the recommendation that fracture risk assessments be undertaken for all aged care residents and used to guide treatment without needing a BMD test [4, 14, 16–18]. Multiple fracture risk assessments exist; however, two tools have been specifically developed and validated for use in the aged care population, the Fracture risk assessment in long-term care (FRAiL) and the Fracture Risk Score (FRS) [4, 16, 18].

In recent years, there has been a global trend for pharmacists to undertake non-dispensing services to optimise medicine use for aged care residents [19, 20]. Internationally, medication management reviews are the most frequent non-dispensing service for aged care residents [19, 20]. Medication reviews have been shown to improve the appropriate use of medicines and are endorsed in consensus recommendations on preventing osteoporotic fractures in aged care facilities [4, 19].

In Australia, pharmacists provide medication reviews for aged care residents through the federal government-funded Residential Medication Management Review (RMMR) services programme [20]. This programme is similar to “clinical medication reviews” in the UK, “comprehensive medication reviews” in the USA, and “MedsCheck LTC” in Canada [21, 22]. RMMRs are intended to be a collaborative service that involves accredited pharmacists providing a written report identifying clinical recommendations to the resident’s physician [20, 23]. Regular medication reviews are considered best practice in professional practice standards [23]. Current recommendations advise that a medication review is completed as soon as possible after the resident’s admission to a facility and periodically thereafter [20, 23].

There is a clear need to address the widespread sub-optimal management of osteoporosis amongst aged care residents. A critical first step towards achieving this is understanding how osteoporosis management occurs in real-world clinical practice. This study investigates the prevalence of osteoporosis medicine use amongst aged care residents and explores drug-related problems (DRPs) identified during medication reviews and pharmacist recommendations to resolve them.

## Methods

### Data collection

A retrospective cross-sectional study of de-identified medication review reports was conducted. Sample size calculations determined that 980 RMMR reports would provide a representative sample of aged care residents receiving osteoporosis medicines [24]. This sample size was based on a 99% confidence interval, 5% margin of error, and prevalence of osteoporosis medicine use previously reported, with an allowance for a proportion of reports not to contain all relevant data fields [6–9, 25].

To enhance the robustness of the sample, all RMMR service providers practicing across Australia were invited to participate. A sample of medication review reports was sought, with the number of reports requested from each provider determined by the number of aged care residents they service. Providers were instructed to supply retrospective consecutive reports from 30th June 2022. All reports were de-identified in relation to residents, healthcare providers, and facilities before being provided to the researchers.

### Data analysis

Resident demographics, diagnoses, medications, and pharmacist recommendations were extracted from each report. The International Classification of Diseases, eleventh revision (ICD-11), was used to categorise diagnoses [26]. Medications were categorised according to the World Health Organization (WHO) anatomical-therapeutic-chemical (ATC) classification system [27]. Osteoporosis medicines were defined as medications indicated for the treatment of osteoporosis in the Australian Medicines Handbook, including the antiresorptive therapies alendronate (ATC code: M05BA04, M05BB03), ibandronic acid (M05BA06), pamidronate (M05BA03), risedronate (ATC code: M05BA07) zoledronic acid (ATC code: M05BA08), denosumab (ATC code: M05BX04), and raloxifene (ATC code: G03XC01), as well as anabolic agents romosozumab (ATC code: M05BX06), teriparatide (ATC code: H05AA02), and nutritional supplements calcium (ATC code: A12AA, A12AX) and vitamin D (ATC code: A11CC04, A11C05, A12AX, M05BB03) [27, 28]. Descriptive statistical analysis, including frequencies, mean, and standard deviations, was completed using the SPSS software package [29].

DRPs were classified via an adapted Hepler and Strand classification system appropriate for aged care residents [30, 31]. This system defines DRPs as events or circumstances that actually or potentially interfere with optimal health outcomes [30]. Categories include indication, effectiveness, and safety, each with sub-classifications.

Pharmacist recommendations were included in the analysis if DRPs involved osteoporosis medicines or medications contributing to osteoporosis. These were explored via content analysis with the aid of NVivo Pro 12 [32]. A data-driven coding frame was developed to identify themes amongst the recommendations, using successive summarisation to develop categories as outlined by Schreier [33]. Two rounds of coding were completed on the first 500 reports to develop and pilot the coding frame. Double coding was employed for the main analysis of the complete data set to increase the reliability of the analysis.

One researcher (CL) performed data extraction, coding, and analysis. Throughout data analysis, regular research team meetings were held to discuss the classification of DRPs and the recommendation coding framework.

### Ethics

This study was approved by the Human Research Ethics Committee (HREC) at the University of Technology Sydney (ETH22-7751). All RMMR service providers provided written informed consent.

## Results

A total of 980 RMMR reports for aged care residents from metropolitan, regional, and rural regions of six of the eight Australian states and territories were received. Eleven reports were excluded as they did not contain all data fields, resulting in a sample of 969 medication review reports. Characteristics of residents represented by these reports are presented in Table 1.

**Table 1:** Resident characteristics (*n* = 969)

Demographics	Mean age in years (± SD)	86 (± 8)
	Female	632 (65.2%)
	Resident location by Australian state or territory	
	New South Wales	371 (38.3%)
	Queensland	366 (37.8%)
	Victoria	149 (15.4%)
	South Australia	37 (3.8%)
	Western Australia	32 (3.3%)
	Australian Capital Territory	14 (1.4%)
Osteoporosis medicine use	Vitamin D use	610 (63.0%)
	Calcium use	123 (12.7%)
	Antiresorptive use (total)	211 (21.7%)
	Denosumab	185 (19.1%)
	Alendronate	12 (1.2%)
	Risedronate	13 (1.3%)
	Raloxifene	1 (0.1%)

### Osteoporosis medicines use

Amongst the 969 residents, four resident subgroups were identified: those receiving antiresorptive therapy with a listed diagnosis of osteoporosis (123, 12.7%), those receiving antiresorptive therapy without a listed diagnosis of osteoporosis (88, 9.1%), those with a listed diagnosis of osteoporosis not receiving antiresorptive therapy (129, 13.3%) and those without a diagnosis and not receiving treatment (629, 64.9%). In total 211 (21.7%) residents had an antiresorptive included in their medication list, of which the majority were prescribed denosumab (185, 87.2%), with the remainder using bisphosphonates (alendronate (12, 5.7%) and risedronate (13, 6.2%)) and raloxifene (1, 0.5%). Of the 88 residents receiving antiresorptive therapy without a listed diagnosis of osteoporosis, one was prescribed denosumab at a dosage associated with treating bone metastasis in breast cancer; all others received a dose consistent with osteoporosis diagnosis. Adding these residents to those with a listed diagnosis (252, 26%) raised the prevalence of osteoporosis to 339 (35.0%).

Vitamin D was taken by 610 (63.0%) residents, and 123 (12.7%) residents received calcium. Of the 211 residents prescribed an antiresorptive, 168 (80.1%) concurrently received vitamin D and 61 (28.9%) used calcium.

### Drug-related problems and recommendations

The 969 medication review reports identified 2637 DRPs with an average of 2.7 DRPs (± 1.5) per report. Osteoporosis-related DRPs represented 368 (14.0%) of these and were included in 348 (35.9%) reports. Most DRPs involved Vitamin D (204, 55.4%), either alone (174) or in combination with calcium (30). The remaining DRPs involved antiresorptive therapies (86, 23.4%), medications contributing to osteoporosis (60, 16.3%), and calcium alone (18, 4.9%). DRPs and associated recommendations relating to osteoporosis medicines are presented in Table 2.

**Table 2:** Drug-related problems and recommendations relating to osteoporosis medicines

Drug-related problem		Summary of DRP and recommendation (*n*)
Indication	Need for an additional drug: undertreatment for diagnosis	Vitamin D and/or calcium: - Vitamin D deficiency is common in aged care residents. Commence 1000iU daily (24) - Resident at high risk of fracture. Commence calcium supplement (3) Antiresorptive therapy - History of osteoporosis, not receiving antiresorptive therapy. Commence antiresorptive therapy (18)
	Need for diagnostic test: unclear or not confirmed indication; need for review	Vitamin D and/or calcium - Vitamin D deficiency is common in aged care residents. Review serum level and supplement if required (27) - Resident at high risk of falls and fractures. Review serum level to guide vitamin D dose (24) - Resident at high risk of fracture. Review calcium dietary intake; if insufficient, commence calcium supplement (12) Antiresorptive therapy - History of minimal trauma fracture, not receiving antiresorptive therapy. Measure BMD via DEXA with a view to commencing therapy (14) - History of osteoporosis, not prescribed antiresorptive therapy. Evaluate fracture risk with a view to commencing therapy (10) - At risk of osteoporosis. Measure BMD via DEXA with a view to commencing antiresorptive therapy (3) - History of osteoporosis, not receiving antiresorptive therapy. Review past medical history and commence antiresorptive therapy if not previously used (1)
	Unnecessary treatment: no appropriate medical indication; therapeutics or pharmacological duplication; drugs used for the treatment of avoidable adverse drug reactions	Vitamin D and/or calcium - Vitamin D provides no clinical benefit in individuals without frank deficiency. Cease vitamin D (19) - Resident at end of life. Cease vitamin D (11) - Vitamin D is replete per serum level. Cease vitamin D (5) - Calcium is only of benefit if dietary intake is insufficient. Review dietary intake, if sufficient, cease calcium (4) - Need for calcium is negated by concurrent use of vitamin D. Cease calcium (3) - Resident is non-ambulating and hence has a low fracture risk, calcium provides limited clinical benefit. Cease calcium (2) - Resident taking multiple medications. To reduce polypharmacy, cease calcium (1) - Resident at end of life. Cease calcium (2) Antiresorptive therapy - Resident has low falls risk. Cease denosumab (2) - Resident at end of life. Cease bisphosphonate (2) - Resident at end of life. Cease denosumab (1)
Effectiveness	Choice of drug: drug not indicated for condition; more effective drug available; contraindication present	Vitamin D and/or calcium - Vitamin D only provides clinical benefit if combined with calcium. Change vitamin D supplement to vitamin D/calcium (36) - Complex medication regimen. To reduce pill burden and improve adherence, change to vitamin D and calcium to a combination formulation (19) - Resident experiencing swallowing difficulties. Change dosage form to improve adherence (6) - Cholecalciferol conversion to active form is impaired by renal impairment. Change cholecalciferol to calcitriol (3) Antiresorptive therapy - Currently receiving raloxifene, other antiresorptive therapies are more effective. Change to denosumab. (1) - Bisphosphonate therapy is contraindicated when CrCl < 30 mL/min, which is the case for this resident. Cease bisphosphonate. (1)
	Dosage too low	Vitamin D and/or calcium - Vitamin D dose < 1000iU daily. Increase to 1000iU daily (4) - Vitamin D is included on the medication list but not being administered. Consultation with care staff to ensure treatment is received (2) Antiresorptive therapy - Previously prescribed denosumab, however, therapy has been disrupted. Chart review and consultation with care staff to ensure timely administration. (9) - Medical history references annual zoledronic acid injections, but this medication is not listed. Chart review and consultation with care staff to ensure timely administration. (1)
Safety	Risk for single adverse drug reactions: unfavourable safety profile	Vitamin D and/or calcium - Resident experiencing constipation, which may be exacerbated by calcium. Cease calcium (5) - Resident is hypercalcaemic. Cease calcium (1) Antiresorptive therapy - Denosumab use is associated with hypocalcaemia. Monitor vitamin D and calcium serum levels and ensure they are replete prior to each dose of denosumab. (32) - Bisphosphonate therapy is associated with hypocalcaemia. Review calcium and vitamin D and supplement if indicated. (2) - History of allergic reaction to denosumab. Revise medication allergies to prevent re-exposure. (1)
	Drug-drug interaction	Vitamin D and/or calcium - Other medications absorption can be impeded by calcium. Adjust the administration regimen to separate the dosing of calcium and the affected medication (2) - Concurrent use of vitamin D and denosumab, experiencing hypercalcemia. Cease vitamin D (1) Antiresorptive therapy - Nil
	Dosage too high; excessive treatment duration	Vitamin D and/or calcium - Vitamin D > 1000iU daily, serum level shows replete. Reduce to 1000iU daily (13) - Vitamin D > 1000iU daily. Complete serum level with a view to reducing dose (13) - Vitamin D > 1000iU daily. Likely to be corrected, reduce dose to 1000iU daily (11) - Calcium > 600 mg daily. Reduce to 600 mg daily (5) - Vitamin D 1000iU daily on the medication list, however, care staff administering 2000iU daily. Consultation with care staff to ensure 1000iU daily administered (2) - Vitamin D > 1000iU daily. Likely to be corrected, change to high-dose intermittent dosing (1) Antiresorptive therapy - Bisphosphonate therapy duration > 5 years. Cease bisphosphonate. (2)

DRPs involving vitamin D and/or calcium were mostly concerned with pharmacists identifying potential under and overtreatment. Individual pharmacists made conflicting recommendations regarding the need for vitamin D. For instance, 24 medication reviews advised that vitamin D should be commenced as deficiency is common amongst aged care residents. In contrast, 19 recommendations advised the cessation of vitamin D as it provides no clinical benefit in individuals without frank deficiency. Where references were provided for these recommendations, proponents of vitamin D use cited aged care-specific guidelines [4]. In contrast, those advocating the cessation of vitamin D cited literature that was not aged care-specific [34–37]. Furthermore, there was inconsistency in the target vitamin D level advised in different medication reviews. The consensus recommendations for the prevention of osteoporotic fractures in aged care advise that the optimal vitamin D level is > 75 nmol/L; the target level suggested in some reports was as low as > 25 nmol/L [4]. Similarly, different pharmacists made conflicting recommendations regarding the use of calcium. In 36 medication reviews, it was recommended that calcium be added to vitamin D supplementation without evaluating dietary calcium intake on the premise that vitamin D is only beneficial if combined with a calcium supplement. In other medication reviews, pharmacists recommended cessation of calcium, citing that calcium supplements are only beneficial when dietary intake is insufficient.

As shown in Table 2, pharmacists identified DRPs involving antiresorptive therapies in the three categories. Indication was the most frequent DRP category, with most recommendations advocating the commencement of antiresorptive therapy. An emphasis on obtaining BMD to guide therapy was seen in these recommendations. In all cases when the commencement of a specific antiresorptive therapy was advised, denosumab was recommended. Denosumab was also involved in the majority of DRPs concerning effectiveness and safety. The most common DRP relating to effectiveness concerned the dosage being too low due to inadvertent therapy disruptions of denosumab (9) and zoledronic acid (1). The most common safety related DRPs involved the risk of hypocalcaemia and the subsequent need to monitor vitamin D and calcium when administering denosumab (32).

Medications contributing to osteoporosis risk and related recommendations were explored. There were 60 DRPs concerning osteoporosis risk, with the drug class most often involved being proton-pump inhibitors (PPIs) (39, 65.0%), followed by corticosteroids (11, 18.3%), anti-epileptics (4, 6.7%), gonadotropin releasing hormone analogues (2, 3.3%), aromatase inhibitors (2, 3.3%), other hormone antagonists and related agents (1, 1.7%), and thiazolidinediones (1, 1.7%). Ensuring residents had adequate vitamin D and/or calcium intake was advised for all drug classes. Dose reduction or cessation was the most common recommendation to address the risk of osteoporosis from PPIs, corticosteroids, and aromatase inhibitors. Other recommendations involved measuring BMD to guide the commencement of antiresorptive therapy and considering a change of medicine.

## Discussion

This study provides a unique perspective on osteoporosis management amongst aged care residents. The use of osteoporosis medications has been presented alongside an analysis of recommendations made by pharmacists to address DRPs concerning osteoporosis. Identified DRPs and pharmacist recommendations to resolve them highlight aspects of clinical practice that can be targeted to improve osteoporosis management in this setting.

The findings of this study confirm previous reports that osteoporosis is underdiagnosed in aged care residents. The finding that 35.0% of residents had osteoporosis is consistent with a previous Australian study that reported 34.1% of residents had a documented osteoporosis diagnosis [5]. However, this is significantly lower than the estimated 80–85% prevalence rate based on BMD testing of aged care residents [2]. A potential contributing factor to underdiagnosis is that of incomplete medical histories being provided to aged care facilities at the time of resident admission [10]. The finding that 41.7% of residents receiving antiresorptive therapy did not have osteoporosis in their diagnosis list supports the notion that incomplete medical histories are a common occurrence for aged care residents.

Antiresorptive therapies were used by 21.4% of all residents and 48.8% of those with osteoporosis as a listed diagnosis. These usage rates, although still indicative of undertreatment, are higher than those reported in previous studies, which have been as low as 4.5% of all residents and 30% of residents with a documented diagnosis of osteoporosis [6, 7]. The higher use of antiresorptive therapies found in this study may be attributed to the rising popularity of denosumab. Previously, oral bisphosphonates have been the predominant antiresorptive agent, although increasing use of denosumab was found between 2014 and 2017 [6–9]. In this study, denosumab was the most frequently used antiresorptive, being used by 87.1% of residents receiving therapy. Australian clinical guidelines and consensus recommendations support a preference for subcutaneous denosumab or intravenous zoledronic acid for aged care residents, given the complex administration requirements and adverse effect profiles of oral bisphosphonates [4, 14]. Whilst denosumab and zoledronic acid are equally effective, zoledronic acid is contraindicated in those with reduced renal function (eGFR < 35 mL/min), and the administration requirements of denosumab are more conducive to the aged care setting [4, 18]. The predominant use of denosumab observed in this study indicates these clinical guidelines and consensus recommendations are being adopted in clinical practice, however undertreatment persists.

Pharmacists frequently identified undertreatment of osteoporosis as a DRP for residents with a documented history of osteoporosis or minimal trauma fracture. However, in one-third of these cases, pharmacists recommended measuring BMD via DEXA to determine if an antiresorptive should be commenced. This is despite evidence that obtaining BMDs can be extremely difficult for aged care residents and is not necessary for the commencement of antiresorptive therapy in those with established osteoporosis, including a clinical history of minimal trauma fracture [4, 10, 11, 14, 16, 17]. Furthermore, although there appeared to be a high level of underdiagnosis of osteoporosis amongst residents receiving a medication review, pharmacists were not proactive in raising this. These findings highlight a need to develop and implement clinical practices and protocols to ensure all residents who would benefit from antiresorptive therapy are identified. One strategy that may facilitate this is incorporating the FRS or FRAiL as a routine part of the medication review process. Aged care-specific clinical guidelines and consensus recommendations advocate vitamin D for all residents, except those at end of life [4, 14, 17]. This widespread use of vitamin D is recommended due to high rates of vitamin D insufficiency amongst aged care residents [4, 14, 17, 38]. In this study, Vitamin D was used by 63.0% of residents, which is at the higher end of vitamin D use by aged care residents reported in other studies (16.2–64.0%) [5–7, 25, 38–40]. The higher rate of vitamin D observed in this study suggests uptake of these clinical guidelines and recommendations. However, exploration of pharmacists' recommendations revealed that, like other healthcare professionals, they hold mixed views on vitamin D use [10, 11, 39]. Sometimes these views translated into recommendations inconsistent with aged care-specific guidelines and consensus recommendations. Of note, 19 medication review reports advised stopping vitamin D for residents not at the end of life. These findings are consistent with previous research, which found pharmacists undertaking medication reviews focus on deprescribing opportunities, including vitamin D [10]. Accordingly, a continuing need to raise awareness amongst healthcare professionals in aged care about the benefits of vitamin D supplementation is clear.

Adequate vitamin D and calcium intake is critical for those residents receiving antiresorptive therapy for two reasons: to reduce the risk of hypocalcaemia and optimise the clinical effect of antiresorptive therapy [4, 14, 17, 28]. In the case of injectable antiresorptive therapies, such as denosumab, the risk of hypocalcaemia is highest immediately after administration [14, 28]. Clinical guidelines advise that calcium and vitamin D serum levels be tested before each dose and corrected before administration of the antiresorptive [14, 17, 28]. Pharmacists frequently flagged the need for this monitoring in their recommendations concerning denosumab, suggesting it is often omitted in clinical practice. Furthermore, of residents receiving antiresorptive therapy, 19.1% did not concurrently take vitamin D, and only 28.9% received calcium. These findings suggest a need for greater education and interprofessional collaboration amongst those involved in caring for aged care residents to ensure indicated vitamin D and calcium monitoring and supplementation occurs.

For those residents receiving vitamin D and calcium supplements, recommendations concerning adherence were frequent. Primarily, these recommendations sought to reduce the complexity of the residents’ medication regimen. In recent years, medication regimen simplification has been identified as a way of reducing adverse health outcomes that result from polypharmacy and associated complex medication regimens, which are common in aged care residents [41]. Medication regimen simplification refers to the process of reducing the complexity of a medication regimen without changing therapeutic intent [41]. This can be achieved, without changing the therapeutic intent, by addressing factors such as the number of medications administered and special instructions for medication administration, for instance, crushing tablets [41, 42]. In this study, pharmacists frequently recommended vitamin D/calcium combination formulations instead of two separate products. Pharmacists also recommended changing vitamin D and/or calcium dosage formulations to improve suitability for residents with swallowing difficulties. These findings highlight the importance of regular medication review to ensure the optimal medication regimen, based on the resident's individual needs, is used for osteoporosis management.

DRPs relating to adherence were also identified for those residents receiving antiresorptive therapies. In several medication reviews, pharmacists identified that treatment with injectable antiresorptive therapies (denosumab and zoledronic acid) had been inadvertently disrupted. This is consistent with previous reports of therapy disruptions being problematic with injectable antiresorptive therapies due to their intermittent dosing regimen and the incomplete transfer of medical records upon a resident's admission [10]. Such disruptions may have minimal impact on fracture risk reduction of aged care residents receiving zoledronic acid, as the BMD effects of a single dose persist for several years [43]. However, these disruptions are highly problematic for residents receiving denosumab, due to its lack of residual effect and associated rise in fracture risk if doses are delayed or missed [44, 45]. Considering the rising prevalence of denosumab use in this setting, a need to develop and implement protocols to ensure the timely administration of denosumab is apparent.

## Limitations

Some limitations must be acknowledged. Firstly, the study cohort is limited to residents who received a medication review. It has been reported that the medication review service is underutilised, with only 49.7% of Australian aged care residents receiving a medication review within 24 months of admission [46]. The possibility exists that differences may be present between residents who do and do not receive medication reviews that prevent the study results from being representative of the entire Australian aged care resident population. Additionally, the study design relied on medication review reports as the sole source of information regarding the residents. Hence, the accuracy of the analysis depended on pharmacists correctly reporting residents' diagnoses and medicines.

## Conclusion

Evidence of osteoporosis underdiagnosis and undertreatment amongst aged care residents was found. This is highlighted by only 26% of residents having a listed diagnosis of osteoporosis, of which less than half received antiresorptive therapy. Pharmacist identified DRPs and recommendations revealed common aspects of clinical practice that can be addressed to improve osteoporosis management for aged care residents. Deviations from aged care-specific clinical guidelines and consensus recommendations concerning vitamin D and calcium highlight an ongoing need for the education of healthcare professionals to ensure these are implemented in clinical practice. The rising popularity of denosumab has created an urgent need to develop and implement facility protocols and procedures to ensure its safe and effective use.
